# Supplementary material for: Research policies and scientific production: A study of 94 Peruvian universities
Source: PLoS One. 2021 May 28;16(5):e0252410. doi: 10.1371/journal.pone.0252410 (PMC8162649; doi:10.1371/journal.pone.0252410)
Supplement: Attachment — Submitted filename: Response to Reviewers.docx [file pone.0252410.s001.docx]

**AUTHOR REPLY FORM**

| Reviewer Number | Comments of the reviewer | Reply by the author(s) | Changes made and page number: |
| --- | --- | --- | --- |
| **1** | An improvement of this article is adding a section about the literature review of the relevant publications (books, scholarly articles and any other sources) relevant to this topic. In this case, as a recommendation, you may have a look at the article: Acosta Roa, E. R., Marin Velasquez, T. D., & Gonzales Caycho, A. M. (2020). Policies for scientific production in Latin America: Peru, a case study. Revista Ciencias Pedagogicas E Innovacion, 8(1), 62–69. https://doi.org/10.26423/rcpi.v8i1.350. | The reviewer’s suggestion was taken into consideration. | 3,4,7,8 |
| **1** | The choice of using variables from a specific year needs to be explained (for instance, why did the authors choose the scientific production from 2016 as an independent variable instead of using the scientific production from 2018?). | This information has been added in the methodology section. | 10 |
| **1** | Please provide a justification for using this methodology and link it to other studies that used the same method. | The bibliometric studies consider the revision of databases. | 8 |
| **1** | Although the section with the interpretation of the results is lengthy, some coefficients should be explained better (for example, in Table 4 what does it mean the effect of the variable entitled Type of management on the number of RENACYT researchers?) | The table was improved, and the requested explanation was added. | 12-15 |
| **1** | 5) Authors should discuss the study's limitations, not Only the future perspectives | This discussion has been added in the last paragraph of the discussion. | 24 |
| **1** | There are several errors in the text (on page 6, the link with the source of the research policies is not working). Also, please check the punctuation (for example, on page 7 in the paragraph about the classification of research policies) and pay attention to the repetitions (the last paragraph from page 12 is redundant since it has similar ideas presented in the last paragraph from page 11). | The errors have  been corrected. | 7,11,12 |
| **1** | The section with the conclusions seems to be a reiteration of the sections about results and discussion plus several recommendations. An improvement will be to state in a more convincing way the significance of your contribution to scientific research. | This section  has been corrected. | 24,25 |
| **2** | I would like to see more discussion about te importance of the topic at least in Latin America | More discussion  has been added. | 22,23,24 |
| **2** | I think it is key for this paper to say something about the comparison of universities with diverse offer of careers and research possibilities. | Although the reviewer’s suggestion is very important, it was not the objective of the research. | N/A |
| **2** | What is the state of the art in this topic? Why is key for the development of a country to count on good research production, moreover in Peru? | This information has been added to the introduction and discussion. | 3,4,23,24 |
| **2** | some arguments must be included about the pertinence o using linear regression models to test such implicit hypothesis in this study. | This information has been added | 10,11 |
| **2** | References about how similar studies are carried on for other countries would help to better understand the model proposed. The Latin American experience might help, mainly the experience of countries similar to Peru | References have been added. | 25-30 |
| **2** | Some literature review may help to discuss who make the decisions about the research policies and how those selected indicators of funding and human capital represent those decisions | Additional literature  review has been added. | 22,23 |
| **2** | It is not clear for me the data used in the analysis. I know the Sunedu y Concytec provide detailed data for universities about some of the variables. Still I assume the authors may have completed their database with institutional information from the universities studied. | The data linked to the policies were obtained directly from the licensing resolutions of each university, as indicated in the methodology section. | 8 |
| **2** | I would ask about the main sources of the detailed data used about the human capital and research funding policies by each university included in the analysis (Table 1). | This information  has been added. | 12 |
| **2** | Tables must have some reference to the specific data source used, and it is important in each table. | References have been added. | 12-15 |
| **2** | The final discussion is more referred to the policy institutions and authorities which is fine. Still some of these results have also being influenced by the own decisions of individual universities and authorities. | This information has been  added at the end  of the discussion. | 21,23-24 |
