# Supplementary material for: Research policies and scientific production: A study of 94 Peruvian universities
Source: PLoS One. 2021 May 28;16(5):e0252410. doi: 10.1371/journal.pone.0252410 (PMC8162649; doi:10.1371/journal.pone.0252410)
Supplement: Attachment — Submitted filename: Response to Reviewers.docx [file pone.0252410.s002.docx]

**AUTHOR REPLY**

**Reply to the reviewers’ comments.**

| Reviewer Number | Comments of the reviewer | Reply by the author(s) | Changes made on page number: |
| --- | --- | --- | --- |
| **1** | To control for heteroskedasticity, the authors should report if the standard error of the regressions are robust. | Information has been added in the methodology and result sections | 10, 15-18,24,25 |
| **1** | Also, some spelling errors need to be corrected (for example p.10 “firstly”, “secondly”). | This has been corrected as suggested | All document |
| **2** | Some issues I still would like to see in the paper are about the data analysis, to be positive that the model applied here is the most adequated. | Information has been added to the methodology and discussion section | 10, 15-18,24,25 |
| **2** | Also, some policy recommendatios may be useful at the end of the document. | Information has been added in the final part of the discussion section | 26 |
| **2** | And some edition suggestions (e.g., in the Reference section) I made before, may help in the final version. | This has been corrected as suggested | 28-33 |
